# Biologically Effective Dose and Rectal Bleeding in Definitive Proton Therapy for Prostate Cancer

**DOI:** 10.14338/IJPT-21-00007.1

**Published:** 2021-09-08

**Authors:** Ronik S. Bhangoo, Molly M. Petersen, Gabriella F. Bulman, Carlos E. Vargas, Cameron S. Thorpe, Jason Shen, William W. Wong, Jean-Claude M. Rwigema, Thomas B. Daniels, Sameer R. Keole, Steven E. Schild, Yi Rong, Todd A. DeWees

**Affiliations:** 1 Department of Radiation Oncology, Mayo Clinic, Phoenix, AZ, USA; 2 Department of Health Sciences Research, Mayo Clinic, Scottsdale, AZ, USA

**Keywords:** prostate cancer, proton therapy, hypofractionation, SBRT, rectal bleeding

## Abstract

**Purpose and Objectives:**

With increasing use of hypofractionation and extreme hypofractionation for prostate cancer, rectal dose-volume histogram (DVH) parameters that apply across dose fractionations may be helpful for treatment planning in clinical practice. We present an exploratory analysis of biologically effective rectal dose (BED) and equivalent rectal dose in 2 Gy fractions (EQD2) for rectal bleeding in patients treated with proton therapy across dose fractionations.

**Materials and Methods:**

From 2016 to 2018, 243 patients with prostate cancer were treated with definitive proton therapy. Rectal DVH parameters were obtained from treatment plans, and rectal bleeding events were recorded. The BED and EQD2 transformations were applied to each rectal DVH parameter. Univariate analysis using logistic regression was used to determine DVH parameters that were significant predictors of grade ≥ 2 rectal bleeding. Youden index was used to determine optimum cutoffs for clinically meaningful DVH constraints. Stepwise model-selection criteria were then applied to fit a “best” multivariate logistic model for predicting Common Terminology Criteria for Adverse Events grade ≥ 2 rectal bleeding.

**Results:**

Conventional fractionation, hypofractionation, and extreme hypofractionation were prescribed to 117 (48%), 84 (34%), and 42 (17.3%) patients, respectively. With a median follow-up of 20 (2.5-40) months, 10 (4.1%) patients experienced rectal bleeding. On univariate analysis, multiple rectal DVH parameters were significantly associated with rectal bleeding across BED, EQD2, and nominal doses. The BED volume receiving 55 Gy > 13.91% was found to be statistically and clinically significant. The BED volume receiving 55 Gy remained statistically significant for an association with rectal bleeding in the multivariate model (odds ratio, 9.81; 95% confidence interval, 2.4-40.5; *P* = .002).

**Conclusion:**

In patients undergoing definitive proton therapy for prostate cancer, dose to the rectum and volume of the rectum receiving the dose were significantly associated with rectal bleeding across conventional fractionation, hypofractionation, and extreme hypofractionation when using BED and EQD2 transformations.

## Introduction

Prostate cancer is the most common male cancer in the United States with close to 200 000 new cases and > 30 000 deaths annually [1]. External beam radiotherapy is a commonly used definitive treatment for localized prostate cancer. Prostate cancer has a low α/β ratio, which helps provide the rationale for the use of hypofractionated treatment regimens to achieve equivalent disease control compared with conventional fractionated regimens. Advances in image guidance and treatment techniques have allowed for increasing use of hypofractionation. Multiple randomized trials have confirmed the efficacy and safety of hypofractionation [2–4]. The use of extreme hypofractionation in the form of stereotactic body radiotherapy has also become more common in recent years with favorable results [5–7].

Although photon therapy is the most common external beam treatment modality, there is an increasing availability of proton therapy for prostate cancer. The rapid dose falloff at the Bragg peak of proton beam results in decreased dose to healthy tissues [8, 9]. Hypofractionated proton therapy for prostate cancer has shown promising early results [10, 11].

Rectal bleeding is a gastrointestinal toxicity associated with prostate radiotherapy. Although rates of rectal bleeding after proton therapy have been reported to be < 5%, 2 database analyses have shown that intensity-modulated radiotherapy was actually associated with less gastrointestinal toxicity compared with proton therapy in the treatment prostate cancer [12–15]. Better characterization of rectal toxicity experienced in patients receiving proton therapy for prostate cancer is needed to understand these differences and to improve proton treatments. Both increasing the radiation dose to the rectum and increasing the volume of the rectum receiving the dose have been associated with higher rates of rectal bleeding [16]. Limited reports have been published specifically analyzing rectal dose-volume histogram (DVH) parameters for rectal bleeding using hypofractionated radiation regimens (in particular with proton therapy) [17, 18]. Moreover, these data do not apply across dose fractionations commonly used in modern treatment regimens.

There are several commonly used dose fractionations for treating prostate cancer. In clinical practice, radiation oncologists should be able to compare both target and organ-at-risk doses across different regimens. The use of biologically effective dose (BED) calculations [19] would allow such a comparison. Studies have shown prostate BED to be significantly associated with tumor control in the setting of prostate cancer, but evidence for the relationship between organ-at-risk toxicity and the BED remains elusive [20].

To identify rectal DVH parameters that apply across dose fractionations, we present an exploratory analysis of the BED for rectal bleeding in patients treated with proton therapy using conventional fractionation, hypofractionation, and extreme hypofractionation regimens.

## Materials and Methods

### Study Design and Setting

After signing informed consent, patients undergoing proton therapy for prostate cancer were enrolled on a multi-institutional, prospective registry (Proton Collaborative Group 001-09, NCT01255748) [21] in the United States. Data on disease outcomes, toxicities, and patient-reported outcomes were collected. Only patients at our institution were included for the current analysis after local institutional review board (19-007866) approval.

### Patient Selection

Patients receiving proton beam radiotherapy for localized prostate cancer with curative intent from January 1, 2016, to December 31, 2019, were included in the study. Included patients had to have completed ≥ 1 baseline and ≥ 1 additional postradiation Expanded Prostate Cancer Index Composite health-related questionnaire. These questionnaires were administered at baseline, end of radiation, 3 months after radiation, 6 months after radiation, 12 months after radiation, and annually thereafter. A minimum of 2 months of follow-up was required for patients to be included. Patients were excluded if they had node-positive or metastatic disease.

### Radiotherapy

All patients were treated with pencil-beam scanning proton therapy. *Extreme hypofractionation* was defined as any treatment regimen of ≤ 5 fractions and ≥ 6 Gy per fraction. *Hypofractionation* was defined as > 2.5 Gy per fraction for > 5 fractions. *Conventional fractionation* was defined as a dose per fraction of 1.8 to 2 Gy. The dose-fractionation for a patient's treatment was at the discretion of the treating physician. Before simulation, carbon fiducial markers (≥ 3) were placed into the prostate gland under ultrasound guidance. In some patients, a hydrogel rectal spacer (SpaceOAR, Augemenix Inc, Bedford, Massachusetts) was placed between the prostate and rectum at the same time as the fiducial marker placement. Simulation was performed with a pelvic-immobilization device and a rectal balloon. The planning computed tomography was coregistered with the T2 sequence from a planning magnetic resonance imaging (MRI). The clinical target volume included the entire prostate gland and the proximal seminal vesicles (typically, 1-1.5 cm). The entire seminal vesicles may have been included for T3b disease. Treatment was delivered with right and left lateral beams. An optimization target volume was created from the clinical target volume using a margin of 2 to 3 mm posteriorly and 3 mm elsewhere. The constructed optimization target volume included an additional 5 mm in the beam direction distally and proximally because of range uncertainty. The robustness of the plan was typically evaluated for 3-mm setup uncertainty and 3% range uncertainty. Image guidance using kV matching of the fiducial markers was done before each treatment. Rectal dose constraints followed institutional guidelines for conventional fractionation, hypofractionation, and extreme fractionation: the volume that received 65 GY (V65 Gy) ≤ 50% and a dose ≤ 5 cm (D0.5 cm^3^) ≤ 103% for conventional fractionation, V44 Gy ≤ 50% and V61 Gy ≤ 15% for hypofractionation, and V24 Gy ≤ 40% and V33.5 Gy ≤ 15% for extreme hypofractionation. Of note, when applying an α/β ratio of 3 for late rectal toxicity, the higher dose constraints for the hypofractionation regimens are approximately 10% higher than the standard fractionation protocols (BED of 98.5 Gy [65 Gy nominal], 110 Gy [61 Gy nominal], and 108 Gy [33.5 Gy nominal] for standard, hypofractionation, and extreme hypofractionation, respectively).

### Dosimetric Data

Rectal DVH parameters were obtained from the treatment plans. They were computed with Dxx representing dose (xx Gy) to a percentage of the rectum volume and Vxx representing the percentage of the rectum volume receiving at least a given dose (xx Gy). Assuming an α/β ratio of 3 for late rectal toxicity, both the BED—BED = D × {1 + [d/(α/β)]}—and EQD2—EQD2 = D × {[d + (α/β)]/[2 Gy + (α/β)]}—of each rectal DVH parameter were calculated [22, 23]. In comparison, the α/β ratio for prostate cancer is generally found to be 1.5.

### Toxicity

Rectal bleeding toxicity was graded according to the Common Terminology Criteria for Adverse Events (CTCAE, version 5.0) for rectal hemorrhage. All rectal bleeding events were independently verified in the medical record by a physician and graded according to CTCAE guidelines. Rectal bleeding was considered a radiation-related, late toxicity if it occurred ≥ 2 months after the end of radiotherapy.

### Statistical Analysis

Rectal DVH parameters were analyzed at increments of 1 Gy for V_xx_ and at increments of 1% for D_xx_ (≤ 100%). Univariate analysis with logistic regression, via the Wald test statistic, was performed to determine DVH parameters that were significant predictors of grade ≥ 2 rectal bleeding. To ensure a familywise error rate of α = 0.05 for each parameter on univariate analysis, we used a Bonferroni-adjusted experimentwise error rate of 0.01 to determine statistical significance. Youden index was used to determine optimum dichotomous cutoff points for clinically meaningful DVH constraints. Because of varying dose/fractionation schedules complicating nominal dose importance, BED and EQD2 were only considered for toxicity models [24]. Based on statistical significance and clinical interpretability, certain dichotomized DVH parameters: D mean, D40, D45, D50, V45, V50, and V55 were included in analyses as predictors for rectal bleeding. Combining univariate analyses of all clinical data and DVH constraints, a stepwise model-selection criterion based on clinical and statistical significance was implemented to fit a “best” multivariate logistic model for predicting CTCAE grade ≥ 2 rectal bleeding. To assess the validity of our multivariate model, parameters with a 2-sided test *P* <.05 were considered statistically significant.

## Results

### Patient and Tumor Characteristics

Two-hundred forty three (243) patients were included in this study. Median follow-up was 20 months (range, 2.5-40 months). Patient and tumor characteristics are presented in **Table 1**. Most patients were white (227; 93.4%) with an ECOG 0 (198; 81.5%). Median age was 71 years (52-91 years). Median preradiation prostate-specific antigen (PSA) was 6.1. There were 188 patients (77.4%) with cT1-2 cancers; 230 patients (95%) had an MRI, with 27 (11.1%) revealing extraprostatic extension and 5 (2.1%) revealing seminal vesicle invasion.

**Table 1:** Patient and tumor characteristics, n = 243.

**Characteristic**	**No. (%)**	**Without rectal bleeding, No. (%)**	**With rectal bleeding, No. (%)**
Age, y			
Median (range)	71 (52-91)	71 (52-91)	74.5 (59-79)
Race			
White	227 (93.4)	219 (94.0)	8 (80.0)
Other	16 (6.6)	14 (6.0)	2 (20.0)
ECOG			
0	198 (81.5)	190 (81.5)	8 (80.0)
1 or 2	45 (18.5)	43 (18.5)	2 (20.0)
Preradiation PSA			
Median (range)	6.140 (0.3-1289)	6.100 (0.3-1289)	9.2 (1.9-28.08)
Preradiation PSA			
< 10	185 (76.1)	180 (77.3)	5 (50.0)
10–20	44 (18.1)	40 (17.2)	4 (40.0)
> 20	14 (5.8)	13 (5.6)	1 (10.0)
T stage			
T1–T2a	188 (77.4)	181 (77.7)	7 (70.0)
T2b–T2c	32 (13.2)	30 (12.9)	2 (20.0)
T3–T4	22 (9.1)	21 (9.0)	1 (10.0)
Gleason score, total			
6 (group 1)	35 (14.4)	34 (14.6)	1 (10.0)
7 (group 2 or 3)	146 (60)	139 (57)	7 (70)
3 + 4 (group 2)	91 (37.4)	89 (38.2)	2 (20.0)
4 + 3 (group 3)	55 (22.6)	50 (21.5)	5 (50.0)
8 (group 4)	36 (14.8)	35 (15.0)	1 (10.0)
9–10 (group 5)	26 (10.7)	25 (10.7)	1 (10.0)
MRI			
Yes	230 (94.7)	220 (94.4)	10 (100.0)
No	13 (5.3)	13 (5.6)	0 (0.0)
MRI results			
Extraprostatic extension	27 (11.1)	27 (11.6)	0 (0.0)
Seminal vesicle invasion	5 (2.1)	4 (1.7)	1 (10.0)
Androgen deprivation therapy			
Yes	155 (63.8)	147 (63.1)	8 (80.0)
No	88 (36.2)	86 (36.9)	2 (20.0)

**Abbreviations:** ECOG, Eastern Cooperative Oncology Group; PSA, prostate-specific antigen; MRI, magnetic resonance imaging.

### Radiotherapy Characteristics and Outcomes

Dose fractionations are listed in **Table 2**. The number of patients undergoing conventional fractionation, hypofractionation, and extreme fractionation treatment were 117 (48%), 84 (34%) and 42 (17.3%), respectively. When looking at patients who developed rectal bleeding, 70% were treated with conventional fractionation compared with only 30% in those who did not.

**Table 2.** Dose fractionation with biologically effective rectal dose (BED), equivalent rectal dose in 2-Gy fractions (EQD2) transformations.

**Dose fractionation**	**BED, EQD2 for prostate cancer (α/β = 1.5)**	**BED, EQD2 for rectal bleeding (α/β = 3)**	**Total No. (%)**	**Without rectal bleeding, No. (%)**	**With rectal bleeding, No. (%)**
Extreme hypofractionation, Gy/fx					
38/5	230.5, 98.8	134.3, 80.6	42 (17.3)	41 (17.6)	1 (10.0)
Hypofractionation, Gy/fx					
60/20	180, 77.1	120, 72	9 (3.7)	9 (3.9)	0 (0.0)
67.5/25	189, 81	128.3, 77	26 (10.7)	24 (10.3)	2 (20.0)
70/28	180, 77.1	128.3, 77	49 (20.2)	49 (21.0)	0 (0.0)
Conventional fractionation, Gy/fx					
75.6/42	166.3, 71.3	121, 72.6	1 (0.4)	1 (0.4)	0 (0.0)
77.4/43	170.3, 73	123.8, 74.3	8 (3.3)	7 (3.0)	1 (10.0)
78/39	182, 78	130, 78	2 (0.8)	2 (0.9)	0 (0.0)
79.2/44	174.2, 74.7	126.7, 76	106 (43.6)	100 (42.9)	6 (60.0)

**Abbreviation:** fx, fractions.

Ten patients (4.1%) experienced rectal bleeding. All 10 were graded as CTCAE grade 2 and were considered late toxicities. Median time from end of radiotherapy to rectal bleeding toxicity was 8.8 months (6.0-25.4 months).

### Dose-Volume Histogram Analyses

The DVH parameters for the rectum were calculated using BED, EQD2, and nominal dose. At intervals of 1% in volume and 1 Gy for dose, each parameter was tested for association with rectal bleeding. On univariate logistic analysis (UVA), many rectal DVH parameters were significantly associated with rectal bleeding across BED, EQD2, and nominal doses. The UVAs at intervals of 5% in volume and 5 Gy for dose are presented in **Table 3**, and intervals of 10% in volume and 10 Gy for dose are shown in the **Figure**. The UVA demonstrated that, for each DVH parameter, exceeding the dichotomized dose-constraint in the treatment plan (all *P* ≤ .03; **Table 4**) was significant for an increased chance of rectal bleeding. The following clinical variables were also tested for significant association with rectal bleeding via the UVA: age, BMI, white race, ECOG ≥ 1, Gleason score, clinical T stage, androgen deprivation therapy use, 5-α-reductase inhibitor use, and dose fractionation. None of the clinical variables were found to be univariately significant (all *P* > .05). Interestingly, the use of conventional fractionation, in comparison with hypofractionation, demonstrated increased chance of rectal bleeding (odds ration [OR], 3.72; *P* = .05).

**Table 3.** Univariate analysis, with associated P-values based on logistic regression, of rectal bleeding with rectal dose-volume histogram parameters using biologically effective rectal dose (BED), equivalent rectal dose in 2-Gy fractions (EQD2) doses.

	**BED**		**EQD2**		**Total dose**	
	**Median**	***P*** **value**	**Median**	***P*** **value**	**Median**	***P*** **value**
Dmean	12.05	< .01	7.23	< .01	8.08	< .01
D0	132.09		79.25		72.07	.01
D5	87.04	.03	52.22	.03	53.03	.01
D10	43.41	< .01	26.05	< .01	31.56	< .01
D15	20.09	.01	12.05	.01	16.51	< .01
D20	9.77	.02	5.86	.01	8.79	< .01
D25	4.99	.02	2.99	.02	4.63	.01
D30	2.79	.03	1.68	.03	2.69	.03
D35	1.73	.04	1.04	.04	1.7	.03
D40	1.25	.03	0.75	.03	1.23	.03
D45	0.932	.03	0.56	.03	0.92	.03
D50	0.72	.05	0.43	.05	0.71	.05
D55	0.55		0.33		0.54	
D60	0.4		0.24		0.4	
D65	0.29		0.17		0.29	
D70	0.202		0.121		0.2	
D75	0.13		0.08		0.13	
D80	0.07		0.04		0.07	
D85	0.03		0.016		0.03	
D90	0.007		0.004		0.007	
D95	0.001	.01	0.001	.01	0.001	.01
D100	0		0		0	
V10 Gy	19.8	.02	16.41	.02	19.03	.02
V15 Gy	17.12	.02	13.48	.02	15.92	.02
V20 Gy	15.04	.02	11.93	< .01	13.47	.02
V25 Gy	13.48	.02	10.25	< .01	11.82	< .01
V30 Gy	12.48	< .01	8.91	< .01	10.42	< .01
V35 Gy	11.59	< .01	7.99	< .01	9.01	< .01
V40 Gy	10.41	< .01	7.21	< .01	7.81	< .01
V45 Gy	9.69	< .01	6.14	.01	6.74	< .01
V50 Gy	8.91	< .01	5.38	< .01	5.72	< .01
V55 Gy	8.2	< .01	4.55	.01	4.48	< .01
V60 Gy	7.81	< .01	3.92	.01	3.36	< .01
V65 Gy	7.4	< .01	3.04	.01	2.39	< .01
V70 Gy	6.77	.01	2.07	.01	0.7	< .01
V75 Gy	6.14	.01	1.01	.04		
V80 Gy	5.74	.01				
V85 Gy	5.21	.01				
V90 Gy	4.74	.01				
V95 Gy	4.16	.01				
V100 Gy	3.92	.01				
V105 Gy	3.42	.01				
V110 Gy	2.79	.01				
V115 Gy	2.24	.01				
V120 Gy	1.64	.02				
V125 Gy	1.01	.04				

Note: α/β = 3

**Figure.**
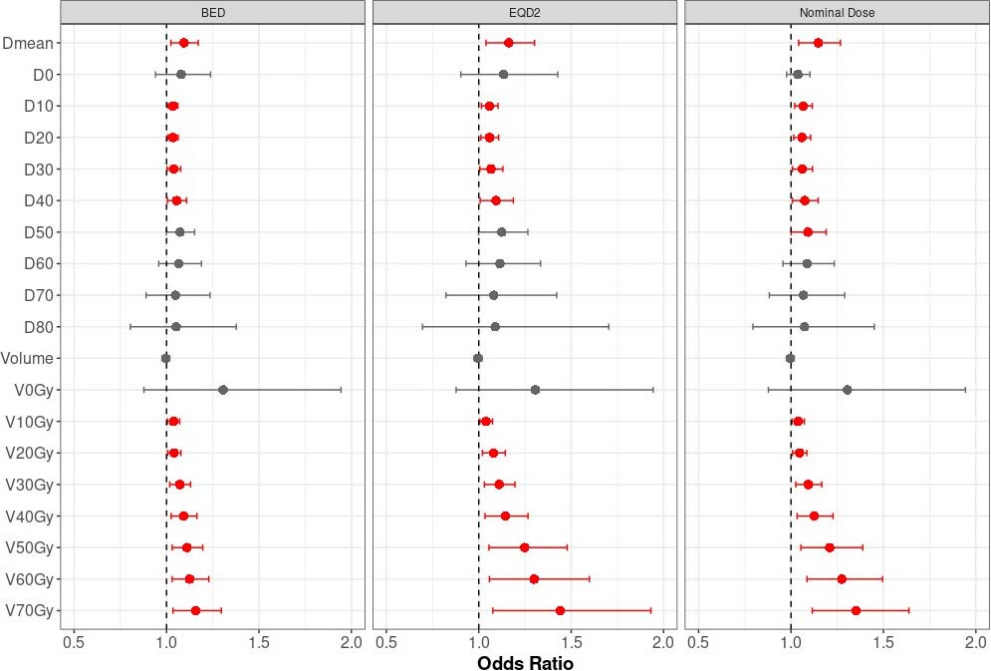
Forest plot of association of rectal bleeding with rectal dose-volume histogram (DVH) parameters by biologically effective rectal dose (BED), equivalent rectal dose in 2-Gy fractions (EQD2), and nominal doses. *An odds ratio (OR) > 1 implies increased risk of rectal bleeding, whereas an OR < 1 implies a decreased risk; an 95% confidence intervals for the OR that include 1 implies statistically nonsignificant effect on rectal bleeding.

**Table 4.** Clinical and dichotomized rectal dose-volume histogram parameters for rectal bleeding.

**Variable**	**OR, No. (range)**	***P*** **value**
Age	1.03 (0.93-1.13)	.62
BMI	0.95 (0.82-1.11)	.5
White	0.04 (0.002-0.64)	.98
Nonwhite	3.91 (0.76-20.18)	.1
ECOG ≥1	1.10 (0.23-5.39)	.9
Gleason		
Group 2	0.76 (0.07-8.70)	.44
Group 3	3.40 (0.38-30.40)	.07
Group 4	0.97 (0.06-16.16)	.75
Group 5	1.36 (0.08-22.81)	.94
Clinical T stage		
≥ T2	0.46 (0.11-1.80)	.26
≥ T3	1.12 (0.14-1.80)	.91
Androgen deprivation therapy	2.34 (0.49-11.27)	.29
5-alpha-reductase inhibitor	3.59 (0.40-32.33)	.25
Extreme hypofractionation (versus conventional fractionation)	2.61 (0.53-12.89)	.59
Hypofractionation (versus conventional fractionation)	2.61 (0.31-21.86)	.66
Cutoff points		
BED		
Dmean ≥ 17.45 Gy	7.2 (1.80-28.79)	< .01
D40 ≥ 1.38%	11.36 (1.42-91.12)	.02
D45 ≥ 0.98%	10.78 (1.34-86.40)	.03
D50 ≥ 0.71%	9.39 (1.17-75.29)	.03
V45 ≥ 15.02%	7.04 (1.76-28.12)	< .01
V50 ≥ 14.53%	7.55 (1.89-30.19)	< .01
V55 ≥ 13.91%	8.12 (2.03-32.52)	< .01
EQD2		
Dmean ≥ 10.47 Gy	7.20 (1.80-28.79)	< .01
D40 ≥ 0.83%	11.36 (1.42-91.12)	.02
D45 ≥ 0.59%	10.78 (1.34-86.40)	.03
D50 ≥ 0.43%	9.39 (1.17-75.29)	.03
V15 ≥ 18.85%	6.44 (1.61-25.67)	< .01
V20 ≥ 17.20%	6.88 (1.72-27.47)	< .01
V25 ≥ 15.47%	6.73 (1.69-26.85)	< .01
V30 ≥ 14.53%	7.55 (1.89-30.19)	< .01

**Abbreviations:** OR, odds ratio; BMI, body-mass index; ECOG, Eastern Cooperative Oncology Group; BED, biologically effective dose; EQD2, equivalent dose in 2 Gy per fraction.

Using the methodology previously described through Youden index, the dichotomization of BED V55 Gy > 13.91% was found to be the most statistically (smallest *P* value) and clinically significant (ability to use in clinical decision-making) parameter for rectal bleeding. This dichotomized dose parameter was subsequently used in all multivariate analyses. Although hypofractionation (hypofractionation or extreme hypofractionation versus conventional fractionation) was nominally significant for rectal bleeding on univariate analysis (*P* = .05), it did not remain significant independently of the inclusion of the BED V55Gy cutoff point (OR, 3.6; *P* = .08). However, the BED V55 Gy remained highly significant, even when controlling for fractionation in the multivariate model (OR, 9.81; 95% confidence interval [CI], 2.4-40.5; *P* = .002).

## Discussion

In patients undergoing definitive proton therapy for prostate cancer, dose to, and volume of, the rectum-receiving dose were significantly associated with rectal bleeding across all dose fractionation schemes when analysis was performed with BED and EQD2 transformations. On multivariate analysis, the BED of V55 Gy remained the only significant factor associated with the development of rectal bleeding.

This study is unique in the application of rectal BED and EQD2 for rectal bleeding across clinically relevant dose fractionations. We demonstrated the significance of rectal DVH parameters at increments of 5% for volume and 5 Gy for dose. These associations held true for both BED and EQD2 transformations. Moreover, when incorporating clinical variables into a multivariate model (including hypofractionation), the selected cutoff value of BED V55 Gy was the only factor significantly associated with rectal bleeding. Comparing rectal dose using these transformations is increasingly important given the multiple radiotherapy regimens used to treat prostate cancer. Moreover, our analysis included extreme hypofractionation, which is increasingly being used in clinical practice.

In an earlier study of conventionally fractionated treatment for prostate cancer, V50 Gy was found to be the most significant factor associated with chronic rectal toxicity [16]. Similarly, a more-recent study of hypofractionation found that a rectal wall V50 Gy ≥ 26% would predict for increased risk of rectal bleeding (along with V32 Gy ≥ 50% and V60 Gy ≥ 10%) [17]. The above 2 studies were done in the context of photon radiation. Our finding of a cutoff point for BED of V55 Gy was consistent with those studies. Another large, single-institution series of > 1,000 patients treated for prostate with proton therapy found that rectal V75 Gy was significantly associated with rectal bleeding [18]. Compared with that study, our study is unique in showing that BED to the rectum is associated with late rectal bleeding, and this potentially may be more helpful during treatment planning given modern dose fractionations.

This study is limited by its retrospective nature and the possibility of selection bias. Although baseline clinical variables were collected, confounding factors not accounted for in this analysis may have been present (eg, bleeding disposition, anticoagulation). However, the purpose of this study was to establish an association of BED and EQD2 DVH parameters with rectal bleeding, which may then be used in future studies and predictive models. Additionally, the rate of rectal bleeding was low (4%)—consistent with prior proton therapy studies [12, 13]. An increased number of events would be preferred from a statistical perspective to further evaluate DVH parameters associated with rectal bleeding.

Another limitation is that the nominal dose of proton therapy does not incorporate relative biologic effectiveness (RBE) and linear energy transfer (LET) effects that may also affect rectal toxicity. Despite an assumed RBE of 1.1 for protons, RBE may be increased at the end of the proton range because of increased LET, which may have an important role in the development of rectal bleeding [25]. These effects may also vary depending on proton technology, beam arrangement, and optimization methods used at individual centers. Given the complexity of this relationship, our institution has recently published on this topic using a novel dose LET volume histogram to predict rectal bleeding [26].

This exploratory study demonstrates that both BED and EQD2 rectal dose are associated with rectal bleeding in the treatment of prostate cancer with proton therapy. Given the increasing implementation of hypofractionation and extreme hypofractionation regimens when treating prostate cancer, further investigations of these parameters in larger patient cohorts with more-comprehensive clinical models would be warranted to develop clinically meaningful planning parameters for proton therapy.
